# Post-discharge Telemonitoring of Physical Activity, Vital Signs, and Patient-Reported Symptoms in Older Patients Undergoing Cancer Surgery

**DOI:** 10.1245/s10434-021-09707-3

**Published:** 2021-02-27

**Authors:** Leonie T. Jonker, Maarten M. H. Lahr, Maaike H. M. Oonk, Geertruida H. de Bock, Barbara L. van Leeuwen

**Affiliations:** 1 Department of Surgical Oncology, University of Groningen, University Medical Centre Groningen, Groningen, The Netherlands; 2 Department of Epidemiology, University of Groningen, University Medical Centre Groningen, Groningen, The Netherlands; 3 Department of Obstetrics and Gynecology, University of Groningen, University Medical Centre Groningen, Groningen, The Netherlands

## Abstract

**Background:**

Postoperative home monitoring could potentially detect complications early, but evidence in oncogeriatric surgery is scarce. Therefore, we evaluated whether post-discharge physical activity, vital signs, and patient-reported symptoms are related to post-discharge complications and hospital readmissions in older patients undergoing cancer surgery.

**Methods:**

In this observational cohort study, we monitored older patients (≥65 years of age) undergoing cancer surgery, for 2 weeks post-discharge using tablet-based applications and connected devices. Outcome measures were post-discharge complications and readmissions; physical activity and patient-reported symptoms over time; and threshold violations for physical activity (step count <1000 steps/day), vital signs (temperature <36°C or >38°C; blood pressure <100/60 mmHg or >150/100 mmHg; heart rate <50 bpm or >100 bpm; weight −5% or +5% of weight at discharge); and patient-reported symptoms (pain score greater than the previous day; presence of dyspnea, vomiting, dizziness, fever).

**Results:**

Of 58 patients (mean age 72 years), 24 developed a post-discharge complication and 13 were readmitted. Measured parameters indicated 392 threshold violations out of 5379 measurements (7.3%) in 40 patients, mostly because of physical inactivity. Patients with readmissions had lower physical activity at discharge and at day 9 after discharge and violated a physical activity threshold more often. Patients with post-discharge complications had a higher median pain score compared with patients without these adverse events. No differences in threshold violations of other parameters were observed between patients with and without post-discharge complications and readmissions.

**Conclusion:**

Our results show the potential of telemonitoring older patients after cancer surgery but confirm that detecting post-discharge complications is complex and multifactorial.

**Supplementary Information:**

The online version contains supplementary material available at 10.1245/s10434-021-09707-3.

## Introduction

Cancer imposes a large burden on global health, predominantly because of the aging population.^1^ In 2018, more than half of new cancer cases and almost two-thirds of cancer deaths occurred in adults aged 65 years and older.^2^ Surgery is often required as a part of the curative treatment of patients with a solid tumor.^3^ Comorbidity and frailty (age-related physiological decline of multiple functions) are common in older patients and increase the risk of developing postoperative complications and being readmitted.^4^ Especially for older patients, postoperative complications and unplanned hospital readmissions have a large impact on their functional recovery, quality of life, and mortality.^5^

Several interventions aimed at decreasing postoperative adverse events have been implemented in oncogeriatric surgery, such as geriatric assessments, preoperative optimization of modifiable risk factors, minimally invasive surgical techniques, and enhanced recovery after surgery programs.^3^ As a result of these interventions, as well as requirements to decrease health care costs and increase capacity, the length of hospital stay (LOS) has been significantly reduced.^6^,^7^ With the shortening of LOS, late complications such as surgical site, urinary tract, and respiratory infections and venous thromboembolic complications can occur in the period after hospital discharge.^8^,^9^ Data on post-discharge complications following oncogeriatric surgery and the circumstances at the time of their occurrence are limited.^8^ Identifying deviations in postoperative recovery at home could possibly support early detection of post-discharge complications, reduce their impact, or even prevent unplanned hospital readmissions.^9^,^10^

Home remote monitoring, or at-home telemonitoring, has been used in a few studies following oncological surgery, to monitor patients’ physical activity, vital signs, and well-being using various types of electronic wearables, activity trackers, mobile applications, symptom surveys, and systems supporting video consultation.^11^–^16^ Although these studies demonstrate that the use of a home monitoring system after oncological surgery is feasible, its effect on clinical outcomes has not yet been demonstrated. In addition, telemonitoring studies focusing on older surgical patients are limited.^17^,^18^ To assess the effectiveness of remote home monitoring in the detection of deviations in postoperative recovery after oncogeriatric surgery, we first need to collect telemonitoring data of oncogeriatric patients with a high risk of postoperative adverse events.

Therefore, we conducted an observational cohort study with the aim of monitoring physical activity, vital signs, and patient-reported symptoms of older patients after hospital discharge following oncological surgery. To do so, we compared characteristics and home monitoring data between groups of patients with and without post-discharge complications and with and without hospital readmissions.

## Methods

### Study Design and Participants

This was a prospective analysis from a single-center observational study with perioperative remote home monitoring of older patients after hospital discharge following oncological surgery in an academic teaching hospital in the north of The Netherlands. Results regarding acceptability and usability of remote home monitoring^19^ and postoperative recovery of physical activity^20^ of the first 50 patients of this cohort have previously been published, as well as the results of the recruitment process of 151 patients of the current cohort.^21^ Patients were eligible for inclusion if they were aged 65 years and older, were scheduled for surgical resection of a solid malignant tumor in the Department of Surgical Oncology or Department of Gynecological Oncology, and had internet access at home. Exclusion criteria were cancellation of surgery, emergency surgery, or perceived incapability to use components of the remote home monitoring system due to contact dermatitis, insufficient understanding of the Dutch language, or severe auditory, visual, cognitive, or ambulatory impairment. The local Medical Ethics Committee approved the study (local registration: 2017/286; Netherlands trial registration: NL8253).

### Remote Home Monitoring

Participants’ physical activity, vital signs, and patient-reported symptoms were measured using commercially available monitoring devices and electronic questionnaires connected to a remote home monitoring system developed within the European Union-funded Connecare consortium (Project Grant Number: 689802). The Connecare system consists of a tablet-based health application for patients, called the self-management system (SMS) and a web-based self-adaptive case management system (SACM) for the care professional. Monitoring data were visible to patients on the SMS and regularly checked by the case manager (research physician) on the SACM. Data were not monitored in real time. Patients were contacted by telephone if data were missing or measurements were outside set values (threshold violations), to provide technical assistance or to obtain additional information regarding parameter deviations. If deemed necessary by the case manager, the treating physician could be contacted. Physical activity was monitored in every patient from the start of the study, and vital signs and patient-reported symptoms were monitored in a subset of patients as the IT system was tested and further developed during study implementation.^19^

### Physical Activity

At preoperative baseline assessment, participants were instructed to wear a commercially available accelerometer-based wearable activity monitor (Fitbit Charge 2, Fitbit Inc., San Francisco, CA, USA). Daily step count was measured preoperatively, in the waiting time between baseline assessment until surgery, and postoperatively during hospital admission on the surgical ward and at home up until 3 months after surgery. Data were transferred via Bluetooth from the activity monitor to the tablet-based Fitbit application and Connecare application. A step count below 1000 was considered a threshold violation, but no step goal was provided to the patient.^22^

### Vital Signs

A subset of the participants was discharged with additional commercially available monitoring devices (Nokia Withings, Issy-les-Moulineaux, France; Connecare SMS) to measure their vital signs every morning for 14 days post-discharge, i.e. temperature, blood pressure, heart rate, and weight. Vital signs were considered abnormal if the temperature was <36˚C or >38˚C, blood pressure was <100/60 mmHg or >150/100 mmHg, heart rate was <50 or >100, or weight was −5% or +5% of weight at hospital discharge.

### Patient-Reported Symptoms

A subset of the participants was asked to complete two electronic health questionnaires in the Connecare application once daily for 14 days post-discharge. The first questionnaire measured pain perception using a horizontal visual analog scale linked to a numerical rating scale, with 0 being ‘no pain’ and 10 being ‘the worst pain imaginable’.^23^ We considered a pain score higher than that of the previous day to be a threshold violation. The second questionnaire was a post-surgical health questionnaire to assess patient-reported symptoms. This consisted of 12 yes/no questions about the presence of problems that might indicate potential complications, regarding (1) breathing, (2) vomiting, (3) dizziness, (4) eating, (5) drinking, (6) urinating, (7) defecating, (8) mobility, (9) fever, (10) resting and sleeping, (11) bathing and washing, and (12) getting (un)dressed. Problems with breathing, vomiting, dizziness, or fever were considered to be alarming symptoms and were counted as threshold violations.

### Data Collection

Patient characteristics on comorbidity, frailty (Groningen Frailty Indicator^24^), (instrumental) activities of daily living,^25^,^26^ nutritional status (Short-Form Mini-Nutritional Assessment^27^), and mental status (Hospital Anxiety and Depression Scale^28^) were collected at the face-to-face baseline assessments. Clinical and surgical data were collected from medical records, including in-hospital and post-discharge complications within 90 days after surgery (as classified by the Clavien–Dindo classification^29^ and the Comprehensive Complication Index^30^), hospital readmission within 90 days after surgery, and timing of post-discharge complications and hospital readmissions. Data that deviated from the post-discharge course were complemented with information gathered by telephone during monitoring and at the 3-month follow-up assessment. Deviations from a normal postoperative course that resulted in consultation with a health care professional but did not require treatment were classified as Clavien–Dindo grade 0.

### Outcome Measures

Outcome measures were post-discharge complications and hospital readmissions, physical activity and symptoms over 14 post-discharge days, and threshold violations of physical activity, vital signs (temperature, blood pressure, heart rate, weight), and patient-reported symptoms.

### Statistical Analysis

Descriptive statistics were used to present baseline and surgery characteristics for patients with and without post-discharge complications and readmissions. Comparison between groups were performed using the independent Student’s *t*-test for continuous parametric data, the Mann–Whitney U test for non-parametric data, and the Fisher’s exact test for categorical data. We presented physical activity and patient-reported symptoms over 14 days. The total of performed measurements and threshold violations per parameter (physical activity, vital signs, and patient-reported symptoms) were presented from the first 14 days after discharge, until hospital readmission, or until study dropout. For physical activity, we also analyzed data from the day of hospital discharge (day 0) and the day before hospital discharge (day −1). The total number of threshold violations per measured parameter are presented, as well as the percentage of patients who experienced one or more threshold violations. We compared physical activity, patient-reported symptoms, and the percentage of patients who experienced one or more threshold violations between the subgroups with/without post-discharge complications and with/without hospital readmissions. A *p*-value lower than 0.05 was considered statistically significant. Data were analyzed using SPSS statistics version 23 (IBM Corporation, Armonk, NY, USA).

## Results

### Enrollment and Dropout

**FIG. 1.**
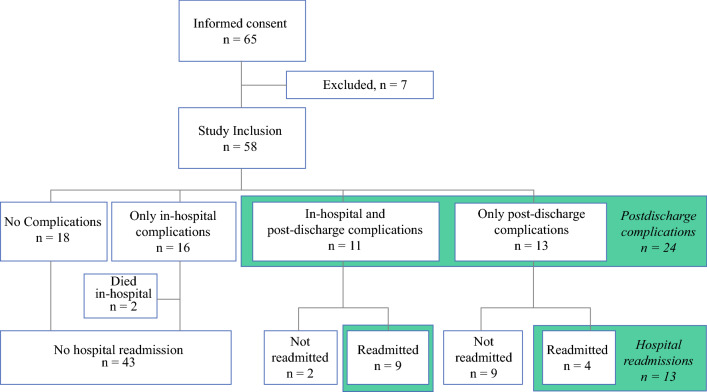
Patients with in-hospital complications, post-discharge complications, and hospital readmissions. After informed consent was obtained, seven patients were excluded from the study because of cancellation of surgery (*n* = 4), missing baseline assessment after rescheduling of surgery (*n* = 2), or regulations regarding the COVID-19 outbreak (*n* = 1). *COVID-19* coronavirus disease 2019

In the period from May 2018 to March 2020, 65 of 130 eligible patients consented to participate in our study. The main reasons for refusal and ineligibility have been extensively described previously.^21^ After informed consent was obtained, seven patients were excluded from the study because of cancellation of surgery (*n* = 4), missing baseline assessment after rescheduling of surgery (*n* = 2), or regulations due to the coronavirus disease 2019 (COVID-19) outbreak (*n* = 1) (Fig. 1). Thus, a total of 58 patients were included in this analysis. After inclusion, 2 patients died and 13 patients withdrew from the study because of the high burden of disease, surgery, or complications in combination with study participation.

### Patient Characteristics

The 58 included patients had a mean age of 72 ± 5 years, and 38 (66%) were male. The majority of patients underwent surgery because of a gastrointestinal malignancy (*n* = 43, 74%), gynecological malignancy (*n* = 5, 9%), or sarcoma (*n* = 4, 7%), with the tumor being intracavitary in 49 (85%) patients. A detailed list of operations performed is presented in electronic supplementary Table A. The median LOS was 8.5 days (interquartile range [IQR] 4.3–19.8). The characteristics of patients with and without post-discharge complications are presented in Table 1.

**Table 1.** Characteristics of patients with and without post-discharge complications

	Patients with post-discharge complications [*n* = 24]	Patients without post-discharge complications [*n* = 32]	*p*-Value
Mean age, years (SD)	72.9 (4.4)	72.1 (5.3)	0.45
Gender			
Male	15 (62.5)	21 (65.6)	
Female	9 (37.5)	11 (34.4)	0.75
ASA classification			
1–2	2 (87.5)	25 (78.1)	
3–4	3 (12.5)	7 (21.9)	0.37
Median Charlson Comorbidity Index (IQR)	3.0 (2.0–6.8)	5.0 (2.0–6.0)	0.37
Location of surgery			
Intracavitary	20 (83.3)	27 (84.8)	
Superficial	4 (16.7)	5 (15.6)	1.00
Surgical technique			
Open	22 (91.7)	22 (68.8)	
Scopic	2 (8.3)	10 (31.3)	0.04*
Median anesthesia time, min (IQR)	378 (187–475)	299 (180–476)	0.56
Median surgical blood loss, mL (IQR)	275 (0–1150)	0 (0–288)	0.09
Median length of hospital stay, days (IQR)	10.0 (4.3–21.8)	8.0 (4.3–15.8)	0.63
In-hospital complications, yes	11 (45.8)	14 (43.8)	0.88
Frail	2 (8.3)	3 (6.3)	1.00
ADL-dependent	1 (4.2)	5 (16.1)	0.22
iADL-dependent	8 (33.3)	7 (21.9)	0.38
Risk of malnutrition	7 (29.2)	7 (22.6)	0.58
Anxiety	3 (12.5)	3 (9.7)	1.00
Depression	10 (41.7)	8 (25.8)	0.21

Data are expressed as *n* (%) unless otherwise specified

Two patients died during hospital admission and were excluded from this table

*SD* standard deviation, *ASA* American Society of Anesthesiologists Physical Status Classification System,^31^*IQR* interquartile range, *ADL* activities of daily living, *iADL* instrumental activities of daily living

* Statistically significant difference, *p* < 0.05

### Postoperative Adverse Events

A total of 40/58 (69%) patients developed a complication within 90 days after surgery: 16 only in-hospital, 11 both in-hospital and post-discharge, and 13 only post-discharge (Fig. 1). Two patients died during hospital admission. Compared with patients without post-discharge complications (*n* = 32), patients with post-discharge complications (*n* = 24) had undergone open surgery more often than laparoscopic or robotic surgery (91.7% vs. 68.8%; *p* = 0.04) (Table 1). The 13 patients who were readmitted had similar patient and surgery characteristics compared with patients who were not readmitted, and they also experienced in-hospital complications more often (9 [69%] vs. 16 [37%]; *p* = 0.04). Table 2 demonstrates that the majority of complications were infectious (*n* = 13, 54%). Most first complications (*n* = 17, 71%) and hospital readmissions (*n* = 8, 62%) occurred within 2 weeks after discharge.

**Table 2.** Details of post-discharge adverse events

Categories and classifications	
Total post-discharge complications	24 (100)
Comprehensive Complication Index, median (IQR)	23.3 (8.7–43.5)
Highest Clavien–Dindo classification complication	
Grade 0	3 (12.5)
Grade 1	8 (33.3)
Grade 2	4 (16.7)
Grade 3A	3 (12.5)
Grade 3B	5 (20.8)
Grade 4A	1 (4.2)
Type of most serious complication	
Infectious	13 (54.2)
‘Failure to thrive’	3 (12.5)
Anastomotic leakage	2 (8.3)
Seroma	2 (8.3)
Thromboembolic event	1 (4.2)
Cardiovascular	1 (4.2)
Drug-induced hypotension	1 (4.2)
False-positive temperature measurement	1 (4.2)
Timing first complication at home	
<14 days after discharge	17 (70.8)
14–30 days after discharge	3 (12.5)
>30 days after discharge	4 (16.7)
Total of patients readmitted	13
Timing hospital readmission	
<14 days after discharge	8 (61.5)
14–30 days after discharge	1 (7.7)
>30 days after discharge	4 (30.8)

Data are expressed as *n* (%) unless otherwise specified

*IQR* interquartile range

### Remote Home-Monitoring Results

Of a total of 5379 measurements that were performed 2 weeks post-discharge, 392 measurements in 40/49 (82%) patients violated the threshold. Most threshold violations were caused by low physical activity and deviations in vital signs, mainly blood pressure (Table 3).

**Table 3.** No. of measurements, threshold violations, and patients experiencing threshold violations

Parameter	No. of measurements	Total no. of threshold violations (%)	No. of patients with threshold violations
Overall	5379	392 (7.3)	40
Physical activity	565	168 (29.7)	27
Vitals	1231	151 (12.3)	25
Temperature	332	29 (8.7)	16
Blood pressure	336	62 (18.5)	15
Heart rate	321	18 (5.6)	7
Weight	248	42 (16.9)	6
Patient-reported symptoms	3583	73 (2.0)	17
Pain	271	30 (11.1)	16
Dyspnea	276	6 (2.2)	3
Vomiting	276	4 (1.4)	4
Vertigo	276	32 (11.6)	5
Fever	276	1 (0.4)	1

### Physical Activity

**FIG. 2.**
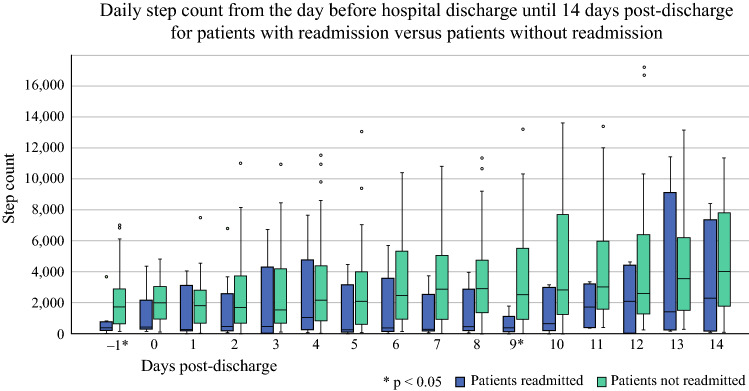
Boxplot of daily step count of patients with readmissions (*blue*) and without hospital readmissions (*light green*) over time. Statistically significant differences were measured on the day before discharge (−1, *p* = 0.01) and on day 9 (*p* = 0.01)

During the first 2 weeks post-discharge, the median daily step count increased from 1600 steps (IQR 500–2930) on day 1 to 3651 steps (IQR 1027–7579) on day 14, without any differences between groups of patients with or without post-discharge complications. The median step count for patients with readmissions was significantly lower than for patients without readmissions on the day before discharge and on day 9 after discharge (Fig. 2). In addition, a threshold violation (step count <1000) was more often measured in patients who were readmitted compared with patients who were not readmitted (7/12 [58.3%] vs. 20/39 [51.3%]; *p* = 0.02). The rates of patients with threshold violations were similar between the groups with and without post-discharge complications (52.9% vs. 55.2%; *p* = 0.25).

### Vital Signs

**FIG. 3.**
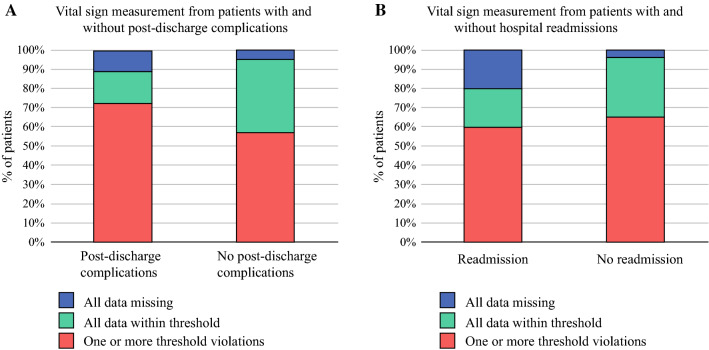
Percentage of patients with threshold violations, values within the set threshold, and data missing for patients with versus without post-discharge complications (**a**) and patients with versus without hospital readmission (**b**). No statistically significant differences were observed

A subset of patients was discharged with a thermometer (*n* = 38), blood pressure/heart rate monitor (*n* = 37), and instructions to manually enter weight into the Connecare application (*n* = 35). A total of 151/1231 vital sign measurements violated the threshold in 25 patients (Table 3). These violations were observed in 13/18 (78%) patients with post-discharge complications and 12/21 (57%) patients without post-discharge complications (*p* = 0.30) [Fig. 3a]. The rates of patients with threshold violations were similar in patients with and without hospital readmissions (Fig. 3b) and per specific vital sign (data not presented).

### Patient-Reported Symptoms

**FIG. 4.**
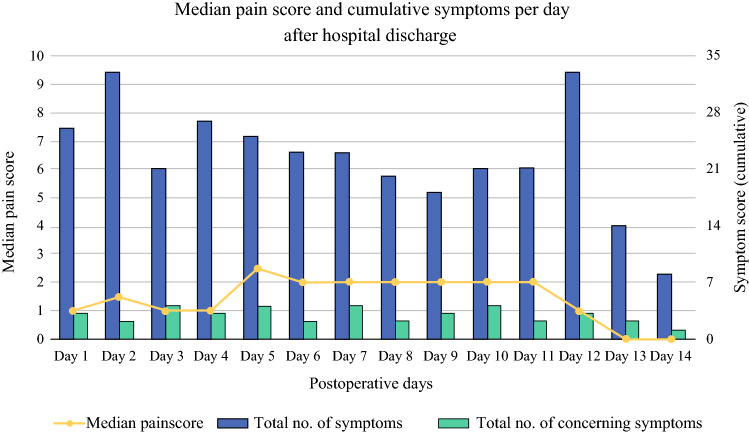
Median pain score and cumulative total of symptoms and alarming symptoms (dyspnea, vomiting, vertigo, or fever) per day after hospital discharge

Thirty-three patients were instructed to report their symptoms. Median pain scores and cumulative symptoms did not change over time (Fig. 4). The pain score over the first 2 weeks was significantly higher in patients with post-discharge complications compared with patients without complications (median 3.0 [IQR 1.9–3.8] vs. 0.5 [IQR 0–1.9]; *p* = 0.02). However, the rates of patients who experienced a threshold violation for pain were similar between groups with and without post-discharge complications (6 [40%] vs. 10 [56%]; *p* = 0.63) and with and without hospital readmissions (3 [38%] vs. 13 [52%]; *p* = 0.13).

The symptoms most frequently reported were needing help with activities of daily living (106 times), being less mobile than usual (78 times), and having trouble sleeping/resting (48 times). In total, 43 threshold violations were caused by nine patients experiencing symptoms of dyspnea, vomiting, vertigo, or fever (Table 3). The percentage of patients who had one or more threshold violations did not differ between patients with and without post-discharge complications (3 [20%] vs. 6 [33%]; *p* = 0.44) or between patients with and without hospital readmissions (2 [25%] vs. 7 [28%]; *p* = 0.25).

## Discussion

In this observational cohort study, we monitored physical activity, vital signs, and patient-reported symptoms of older patients post-discharge after oncological surgery. No evident relation was found between monitored parameters and adverse events, including complications and readmission. To the best of our knowledge, our study is the first to combine remote home monitoring of physical activity, vital signs, and patient-reported symptoms in older patients after cancer surgery; other studies have only combined monitoring of physical activity with symptoms^14^ or vital signs.^12^,^32^

The overall postoperative complication rate in our cohort (69%) is slightly higher than that reported in other studies after oncogeriatric surgery (45–60%),^33^–^35^ but most studies did not report post-discharge complications.^8^ In our cohort, 24 (43%) patients experienced one or more post-discharge complications and 13 (23%) were readmitted, which emphasizes the extent of post-discharge events in this population. Most readmissions were due to infections, but some were due to ‘failure to thrive’, in accordance with data shown in other studies.^8^,^36^ Complications and readmissions occurred most frequently within 2 weeks after hospital discharge,^8^ which would justify the intensive monitoring of various parameters during this period. We expected to detect these complications by measuring a wide range of monitored parameters, but this primarily resulted in a high number of threshold violations, as described previously,^37^ without a clear difference between patients with and without post-discharge events.

Patients who were readmitted had a lower median step count at discharge and in the post-discharge course, and more threshold violations of physical activity compared with patients who were not readmitted. Furthermore, the median pain score of patients with post-discharge complications was significantly higher than that of patients without post-discharge complications. However, the majority of all monitored patients experienced threshold violations of physical activity, vital signs, and patient-reported symptoms in the first 2 weeks after hospital discharge, with similar rates in the groups of patients with and without post-discharge complications and readmissions.

Lower physical activity at discharge was associated with readmissions, in accordance with a previous study where a low inpatient step count resulted in a high risk for 30- and 60-day readmission after metastatic cancer surgery.^38^ Low post-discharge physical activity has already been associated with a complicated postoperative recovery.^14^,^39^ We demonstrated that post-discharge physical activity was lower and more often triggered a threshold violation in patients who were readmitted compared with patients who were not. This supports the idea that postoperative physical activity monitoring could function as an indicator of post-discharge complications;^39^ however, it is unclear whether the complications affect physical activity or the physical activity level elevates the risk of having complications.

Regarding vital sign measurements, we hypothesized to encounter more threshold violations in patients with complications, but no differences between patients with and without post-discharge events were found. In addition, not every patient with a complication or readmission had deviations in vital signs. The absence of threshold violations in case of occurrence of complications could be explained by the fact that most complications were Clavien–Dindo grades 0 and 1 (less severe), and that complications in older patients might not be preceded by deviations in vital signs either.^40^ Furthermore, although our study was solely observational, an interventional monitoring study by Metcalf et al. also found that most vital sign threshold violations did not require an intervention.^12^ Finally, thresholds per parameter were based on standardized values from the early warning scores^41^,^42^ and were not personalized, with the exception of weight loss or gain as a percentage of weight at discharge. If data on patients’ preoperative vital signs are gathered, personalized thresholds could provide a higher sensitivity and specificity to detect complications and readmissions.

The severity and presence of patient-reported symptoms in our cohort did not decline over time, as might be expected based on other studies that monitored symptoms post-discharge after cancer surgery.^14^,^15^,^43^ Although a difference in median pain score between patients with and without post-discharge complications was demonstrated, the use of threshold violations of patient-reported symptoms did not help us to identify patients with post-discharge complications. This could be explained by the fact that our study did not include any feedback or intervention in response to patient-reported symptoms, unlike other studies in which feedback reduced the symptom burden over time.^14^,^15^ The action that was most frequently taken when patient-reported symptoms were present in other studies was reinforcement of prescribed treatment or medication, such as pain medication.^14^–^16^

There are several limitations to our study. Although we aimed to understand the post-discharge recovery in all older patients after cancer surgery, our conclusions were constrained by the data we were able to collect. First, more than half of the identified patients undergoing cancer surgery in our hospital did not participate in the study because of perceived mental or technological barriers.^21^ Second, usability problems, technical issues, study dropout, and variable compliance with performance of measurements resulted in missing data.^19^ The main reason for dropout was a complicated postoperative course in 10 of 13 patients, but the dropout rates in patients with or without post-discharge complications did not differ significantly (29% vs. 19%; *p* = 0.36; not presented in the Results section). The complexity of surgery could also affect the dropout rate, although it was difficult to compare this with each other due to the high variability and the small samples per surgery type. It should be noted that all patients who were approached for participation in this study were planned for complex surgical procedures in a tertiary referral center for oncological surgery. Post-discharge telemonitoring data were only available for 7 of the 13 patients who dropped out, but the median step count was significantly lower on several early post-discharge days than in the general cohort. This could be explained due to a lower compliance to wearing the Fitbit, or by the fact that patients motivated to improve their activity were more motivated to complete the study. Third, not all parameters were measured in all patients at the start of the study as the system was still under development when the additional vital sign and patient-reported symptom monitoring started.^19^ Finally, other important parameters such as respiration rate and oxygen saturation, validated health questionnaires for patient-reported symptoms, and photographs of surgical sites to enable post-discharge wound monitoring^44^ might have contributed more insight into patients’ recovery at home and supported the detection of deviations in recovery.

To address these limitations, future telemonitoring studies should focus on improving accessibility, study inclusion and retention rates, usability, and compliance in older patients after cancer surgery. Our study demonstrates that detecting post-discharge complications following oncogeriatric surgery is complicated and requires more than measurement of a single parameter. Vital sign measurements were not very sensitive or specific for identifying deviations in the post-discharge course of older patients after cancer surgery. It remains to be investigated how this combination affects complication and readmission rates compared with care as usual.

In daily practice, telemonitoring should therefore not be considered a separate tool but rather a supplement to existing perioperative care. In preoperative settings, telemonitoring data could support decision making; for example, whether to start prehabilitation or to proceed with planned surgery. In postoperative care, telemonitoring data should support the existing care for screening, triaging, and scheduling postoperative follow-up. Observed postoperative symptoms could generate automated feedback to patients, which may consist of general nursing advice or early routine or emergency medical consultation and treatment in and outside the hospital. Moreover, post-discharge monitoring after surgery could contribute to better patient–provider communication and promote patient engagement and self-efficacy.^45^–^47^ Perioperative telemonitoring has the potential to improve ‘care as usual’ to personalized and efficient care of the future. Reimbursement of telemonitoring is currently hindered by the ‘fee-for-service’ payment model that stimulates production. In order to incentivize telemonitoring as being part of ‘care as usual’, innovative funding schemes such as bundled payment schemes could be considered.^48^ However, more research on the exact effect on readmission rates and costs in this population is still required.

## Conclusion

Detecting and predicting post-discharge complications is complex and multifactorial. Our results confirm this and provide more insight into which parameters could be used to target post-discharge adverse events after oncogeriatric surgery. Low physical activity and higher pain score were associated with post-discharge events and should be used as parameters in future interventional telemonitoring studies.

## Supplementary Information

Below is the link to the electronic supplementary material.

Supplementary file1 (DOCX 16 KB)
