# Supplementary material for: Post-discharge Telemonitoring of Physical Activity, Vital Signs, and Patient-Reported Symptoms in Older Patients Undergoing Cancer Surgery
Source: Ann Surg Oncol. 2021 Feb 27;28(11):6512–22. doi: 10.1245/s10434-021-09707-3 (PMC7914037; doi:10.1245/s10434-021-09707-3)
Supplement: Supplementary file 1 — Supplementary file1 (DOCX 16 KB) [file 10434_2021_9707_MOESM1_ESM.docx]

## **SUPPLEMENTARY TABLE A** Details of surgery of patients who did and did not complete the telemonitoring study.

| Details surgery (*n* = 58) | | Patients who completed the study  [*n*= 43] | Patients who did not complete the study  [*n*= 15] |
| --- | --- | --- | --- |
| **Type of surgery** | |  |  |
|  | **Intracavitary surgery,** n (%) | **35 (81)** | **14 (93)** |
|  | *Colorectal* |  |  |
|  | - Right hemicolectomy (with/without en-bloc resection involved structures) | 9 |  |
|  | - Pelvic exenteration | 8 | 5 |
|  | - Resection local recurrence colorectal carcinoma | 5 | 2 |
|  | - Rectosigmoid resection (with/without en-bloc resection involved structures) | 3 | 1 |
|  | - Left hemicolectomy (with/without en-bloc resection involved structures) | 1 | 1 |
|  | *Esophago-gastric* |  |  |
|  | - Local resection gastric GIST | 3 |  |
|  | - Subtotal gastric resection - gastric carcinoma | 1 | 1 |
|  | - Esophagectomy | 1 |  |
|  | *Small bowel* |  |  |
|  | - Small bowel resection - NET | 2 | 1 |
|  | *Liver* |  |  |
|  | - Hemihepatectomy | 1 |  |
|  | - Metastasectomy |  | 1 |
|  | *Surgery discontinued due to peritonitis sarcomatosa* |  | 1 |
|  | *Lymph node dissection* |  |  |
|  | - Parailiac | 1 |  |
|  | - Local resection vulva carcinoma + inguinofemoral lymfe node dissection |  | 1 |
|  | **Superficial surgery,** n (%) | **8 (19)** | **1 (8)** |
|  | - Local resection vulva carcinoma + sentinal node biospy | 3 | 1 |
|  | - Lymph node dissection - axillary | 2 |  |
|  | - Superficial lymph node dissection - pelvic | 1 |  |
|  | - Thyreoidectomy | 1 |  |
|  | - Excision sarcoma - extremity | 1 |  |

Patients who dropped out of the study underwent intracavitary surgery more often than patients who completed the study (93% vs. 81%, although statistically not significant (*p* = 0.422). From those patients who did not complete the study, two patients died and 13 patients dropped out

*GIST* gastrointestinal stromal tumor, *NET* neuroendocrine tumor

ss
